# Successful cryoablation for typical atrial flutter in a toddler with sinus node dysfunction: the youngest case report

**DOI:** 10.1093/ehjcr/ytag023

**Published:** 2026-01-28

**Authors:** Mohammad A Ebrahim, Hasan Majid, Mohamed A Abdelnaby, J Philip Saul

**Affiliations:** Department of Pediatrics, Kuwait University Faculty of Medicine, Affiliated with Chest Diseases Hospital, Block 4, Street 102, Jabriya 46300, Kuwait; Department of Pediatrics, Kuwait University Faculty of Medicine, Jabriya 46300, Kuwait; Department of Pediatric Cardiology, Chest Diseases Hospital, Ministry of Health, P.O. Box 4085, Safat 13041, Kuwait; Department of Pediatrics, West Virginia University School of Medicine, PO BOX 9160, Morgantown, WV 26506-9160, USA

**Keywords:** Atrial flutter, Sinus node dysfunction, AV block, Cryoablation, Case report

## Abstract

**Background:**

We describe the youngest (2.8 years, 9 kg) successful cavotricuspid isthmus (CTI) cryoablation whose medical management was complicated with severe sinus node dysfunction and recurrent atrial flutter despite medical therapy (sotalol).

**Case summary:**

Two-catheter, zero fluoroscopy electrophysiology study was performed using EnSite Precision^TM^ mapping system. Cavotricuspid isthmus proved to be ‘in’ circuit with entrainment. Point-by-point 2 min cryolesions resulted in termination of atrial flutter, and later bidirectional block (>120 ms) was confirmed. There was no recurrence post-ablation (>1.5-year follow-up).

**Conclusion:**

Ablation offers a less invasive approach (vs. pacemaker implantation) to toddlers with tachyarrhythmia and coexisting conduction disease, limiting medical management option.

Learning points

- Cavotricuspid isthmus ablation using cryoablation resulted in no recurrence of atrial flutter for at least 1 year of follow-up.
- Ablation offers a less invasive approach (vs. pacemaker implantation) to toddlers with coexisting conduction disease, limiting medical management option.

## Introduction

We describe the youngest (2.8 years, 9 kg) successful cavotricupsid isthmus (CTI) cryoablation in a toddler whose medical management was complicated mainly with severe sinus node dysfunction (SND) and recurrent atrial flutter (AFL) despite medical therapy (sotalol).

Atrial flutter is a macroreentrant atrial tachycardia that incorporates a relatively large reentrant circuit around the cavotricuspid isthmus. It is a relatively rare tachyarrhythmia in children. Paediatric AFL can be associated with structural congenital heart defects and underlying genetic mutations. Because the persistence of such tachyarrhythmia has the potential to cause tachycardia-induced cardiomyopathy, sinus rhythm restoration is the definitive treatment of choice, particularly by ablating the macroreentrant circuit. Nevertheless, sotalol can be used to maintain sinus rhythm in cases not eligible for ablation.

## Summary figure

Timeline of events since presentation until cryoablation.

**Figure:**
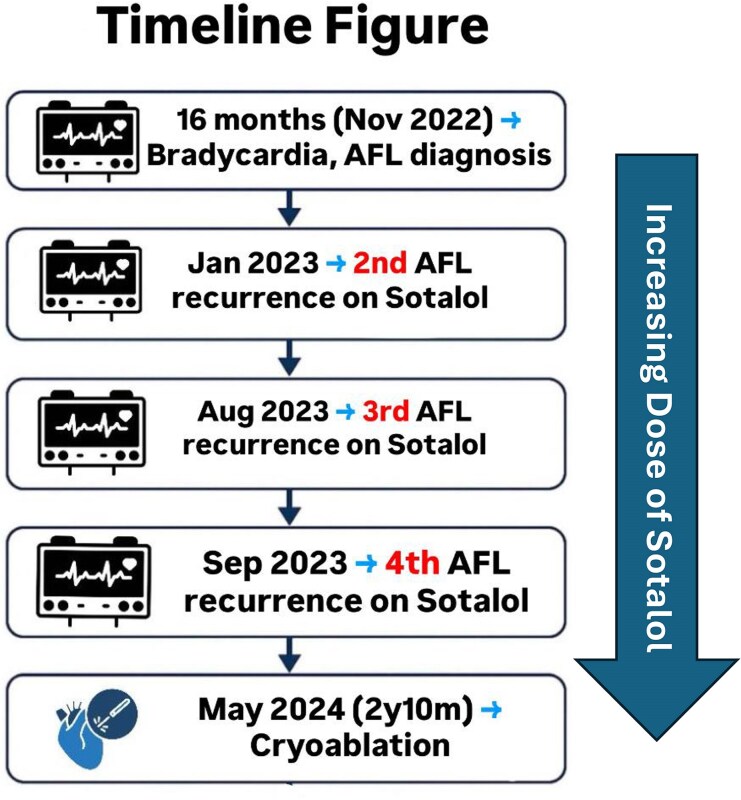


## Case presentation

**Figure 1:**
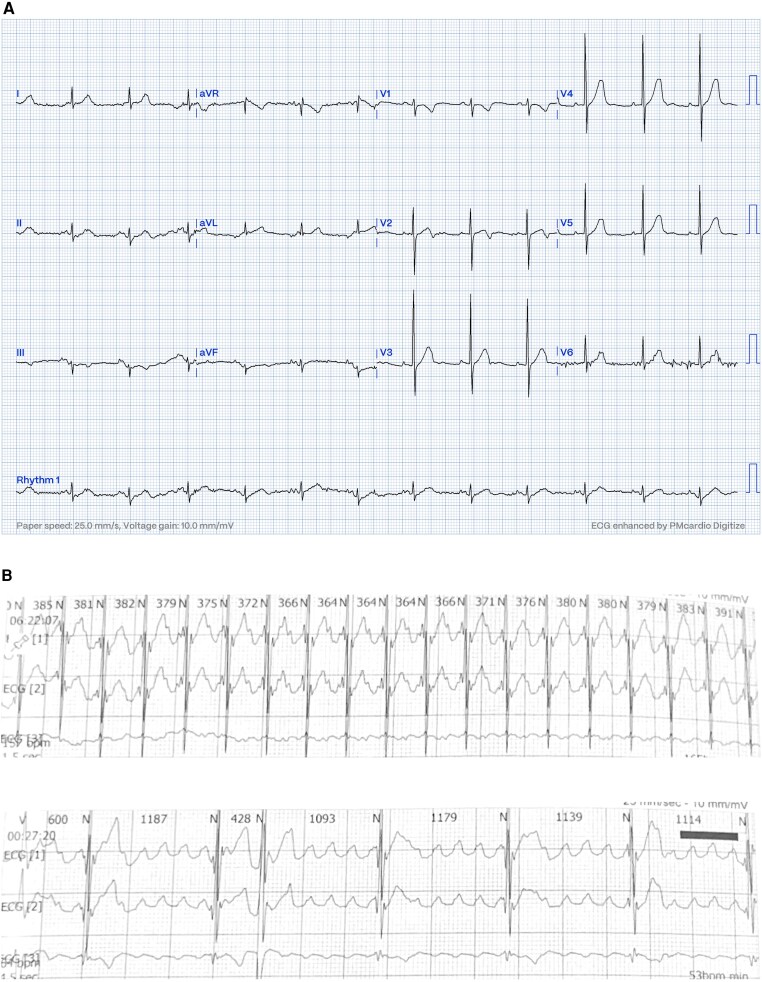
Presenting ECG and initial Holter. (*A*) ECG shows bradycardia, left axis deviation, and negatively deflected P-waves in leads I and AVL suggesting left upper atrial focus. (*B*) Holter ECG shows rapid undulating atrial activity with variable block from as fast as 2:1 conduction producing a heart rate of 150 b.p.m. to as slow as 6:1 conduction producing a pulse rate of 53 b.p.m., with an average heart rate of 92 b.p.m. despite being in flutter and not previously treated with any atrioventricular nodal blocking agent, which suggests an atrioventricular nodal dysfunction alongside a dysfunctional sinus node.

**Figure 2:**
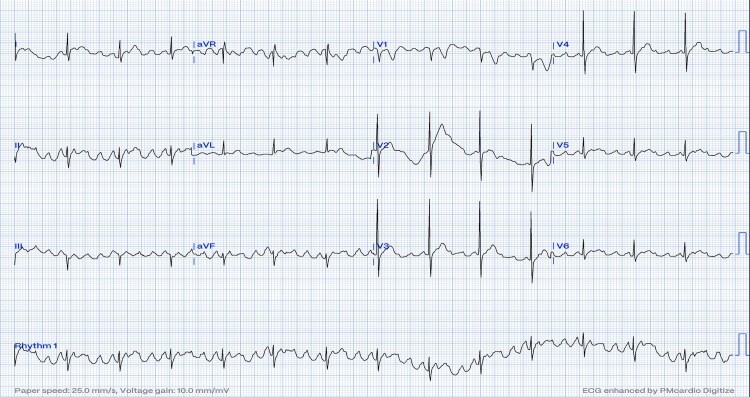
On the first admission. The ECG reveals positive sawtooth-like F-waves in the inferior leads consistent with clockwise cavotricuspid isthmus-dependent atrial flutter at 4:1 conduction.

A 16-month-old child with no past medical or family history was referred for bradycardia at 70 beats per minute (b.p.m.). She was clinically asymptomatic except for poor growth consistent with weight and height below the third percentile. Her presenting electrocardiogram (ECG) (*Figure 1A*) revealed left axis deviation, normal PR interval but with an abnormal P-wave axis, and significant bradycardia indicative of SND. Her echo revealed a small atrial septal defect but was otherwise unremarkable with normal function and dimensions. Unexpectedly, a baseline 24-h rhythm monitoring (Holter) documented persistent AFL throughout (*Figure 1B*), with variable degrees of block (2:1–6:1) and heart rates (HRs) between 53 and 150 b.p.m. The average HR was 92 b.p.m. despite no medical therapy, raising concerns for intrinsic atrioventricular nodal dysfunction. The patient was admitted (*Figure 2*) for transoesophageal echo (TEE) to rule out intracardiac thrombi as the duration of flutter was not known and subsequently was cardioverted. She was started on low-dose sotalol, at 1 mg/kg/day, given the intrinsic conduction disease. Even though the risk of thrombosis in paediatric AFL is low, prophylactic anticoagulation therapy was started after a discussion with the family who preferred starting prophylactic anticoagulation.

Maternal ECG was normal, but a paternal one could not be obtained, and a comprehensive arrhythmia panel (next-generation sequencing) was performed without identifying any abnormal variants.

**Figures 3:**
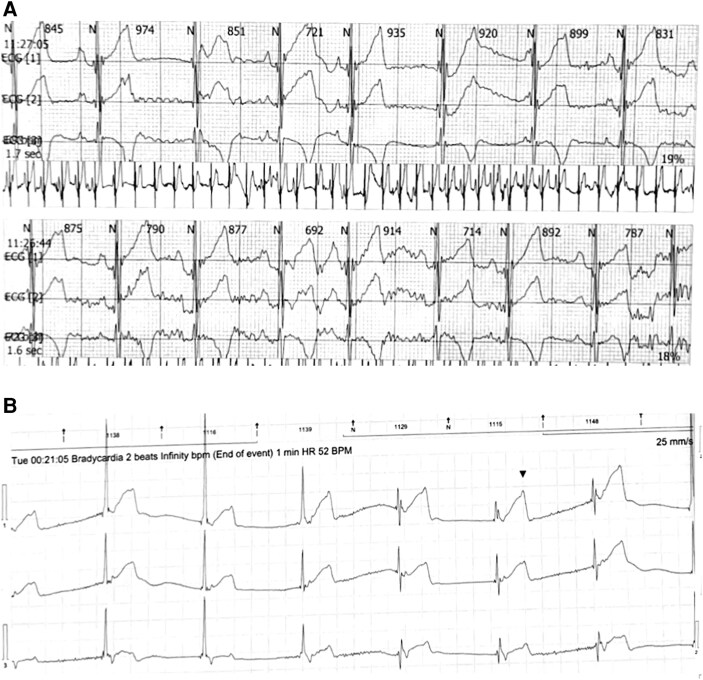
These were recorded after sotalol dose was increased after the second cardioversion. (*A*) Rhythm tracings reveal significant sinus dysfunction with junctional escape. Later sinus rhythm resumes intermittently. (*B*) The QRS complexes here are relatively wider, indicating a ventricular escape origin. The third QRS is narrow and preceded immediately by a sinus beat; thus, this QRS represents a sinus capture beat.

**Figure 4:**
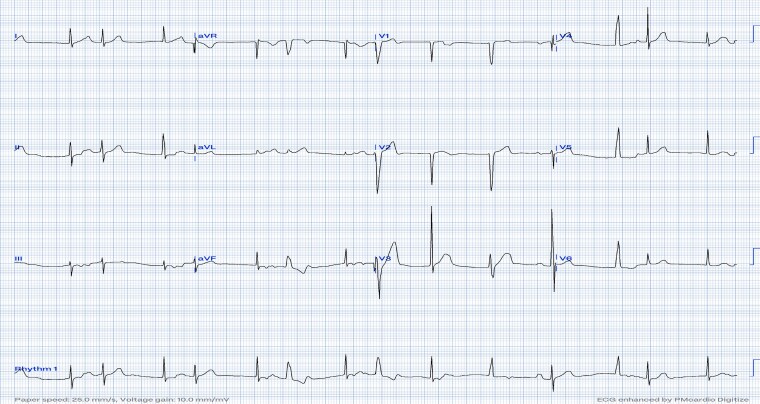
During the third admission after cardioversion. In this ECG, the first beat is not preceded by any atrial activity and is junctional. The second beat is a conducted retrograde P-wave. The third beat appears wider, seen obviously on lead II, indicating a ventricular escape origin. This beat was followed by a retrograde P-wave, best seen in lead III, which was conducted downward to the ventricles producing a narrow QRS complex similar to the first two beats. This phenomenon occurred in the sixth and eighth beats, which were comparatively wider indicating conduction aberrancy. The 13th beat is another atrial capture beat.

Over the next couple of months, the patient had repeated episodes of AFL despite carefully increasing the sotalol dose to a maximum of up to 2 mg/kg/day and was eventually started on anticoagulation. Synchronized cardioversion was successful in terminating each AFL episode after imaging confirmed no evidence for an intracardiac thrombus. Otherwise, follow-up Holters and ECGs showed significant sinus bradycardia with episodes of junctional and ventricular escape rhythms with intermittent sinus capture beats (*Figures 3* and *4*).

**Figure 5:**
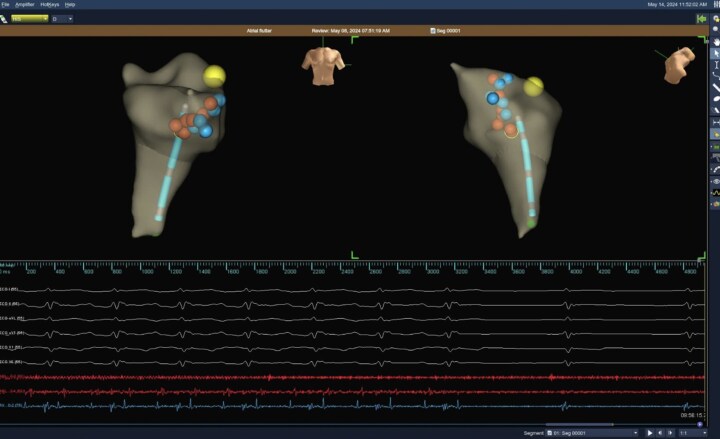
Termination of atrial flutter during cryoablation of cavotricuspid valve isthmus followed by two beats of junctional escape.

The family was offered ablation or pacemaker but initially refused, but eventually agreed for an electrophysiology study (EPS). Warfarin was stopped prior to the procedure with heparin bridge. A 7F right femoral venous access and a 5F left femoral venous access were obtained under general anaesthesia. An EnSite Precision^TM^ mapping system (Abbott, MN) was utilized to allow for a zero fluoroscopy, with 7F Freezor™ Xtra cardiac cryoablation catheter (217F1–49 mm) used initially for mapping and a 5F Supreme Josephson^TM^ quadripolar catheter placed in the right ventricle (Abbott, MN), at 2.8 years (9 kg). The EPS demonstrated CTI-dependent AFL (proved with entrainment), and cryoablation of the isthmus resulted in termination of AFL (*Figure 5*). Linear lesions were created with a total of four point-to-point techniques (2 min per applications) given across the CTI (from the tricuspid valve annulus to the inferior vena cava), with temperatures approaching −80°C. Bidirectional block across the CTI was later confirmed (>120 ms). After ablation and while being off sotalol, the rhythm was initially predominantly junctional rhythm, but with no recurrence of AFL observed over the subsequent 18 months. The patient is now 4 years old (Summary Figure).

## Discussion

Atrial tachyarrhythmia (AT), including AFL, has frequently been reported to coexist with SND.^1^ The bradycardia in SND increases the probability of atrial ectopy due to loss of the protective overdrive suppression over the potential subsidiary pacemaker cells.^1^ On the other hand, prolonged or frequent episodes of AT have been reported to produce structural alterations that sustain the AT, including apoptosis and fibrosis, which electrically decouple the anastomosing cardiomyocytes.^1^ Concomitant electric remodelling may downregulate the inward calcium current and enhance inward rectifier potassium currents, resulting in shortened refractoriness, which sustains the reentrant circuit.^1^ Defective intracellular calcium handling can also lead to increased diastolic intracellular calcium concentrations and increased risk of ectopy.^1^ In some cases, one factor causes or complicates the other by atrial electroanatomic and electrophysiologic remodelling, while in others, a primary atrial cardiomyopathy or inherited channelopathy produces both SND and atrial tachydysrhythmia.^1^ Specifically, mutations in the SCN5A, GNB5 and HCN genes are known to cause SND.^2–4^ Furthermore, loss-of-function mutation in NKX2-5 has been associated with secundum atrial septal defects, SND, and atrioventricular node conduction defects.^5,6^ Otherwise, SND can be associated with complex congenital heart disease or subsequent to cardiac surgery.^7^

Conversion to and maintenance of normal sinus rhythm in patients with AFL is preferable to a rate-control strategy since it relieves the patient’s symptoms, lowers the risk of tachycardia-induced cardiomyopathy, and reduces the risk of systemic thromboembolic events attributable to the AFL.^8^ In patients with concurrent SND, pharmacologic cardioversion is limited by worsening of preexisting SND. Hence, pacing with proper medication dosing or ablation options are useful and were both presented to the family. Normalizing HR through pacing may inhibit AFL development and allow for appropriate dosing of medications. However, given the age of the patient, an epicardial pacemaker would have been the most appropriate option, requiring a surgical approach. Hence, the family elected for ablation to prevent AFL development and to monitor the HR off medications.

Existing recommendations state that ablation can be useful in patients below 15 kg but that medical therapy should be considered prior.^9^ Indeed, medical therapy was complicated with baseline bradycardia. Ablation in the young carries higher risk of mainly vascular complications along with cardiac perforation and coronary artery injury.^10^

Pacing for SND is the only effective modality when symptomatic and all the reversible causes are excluded, but our patient had concurrent AFL.^1^ Given the size and age of the patient, cryoablation was thought to be the safest approach for this patient and perhaps less risk for coronary artery injury compared to radiofrequency approach.^11^ Cryo-lesions are well-defined fibrotic lesions that do not disrupt the ultrastructure of collagen; thus, cryoablation is less likely to damage or cause persistent luminal stenosis of the adjacent blood vessels.^12^

In conclusion, CTI ablation using cryoablation resulted in no recurrence of AFL for at least 1.5 year of follow-up without complications. To our knowledge, this is the youngest report for successful CTI cryoablation. Ablation offers a less invasive approach (vs. pacemaker implantation) to toddlers with coexisting conduction disease, limiting medical management option. The treatment allowed for withdrawal of anti-arrhythmic medication and anticoagulation therapy with avoidance of pacing. The patient continued to be asymptomatic on follow-up with normal cardiac function.

## Lead author biography


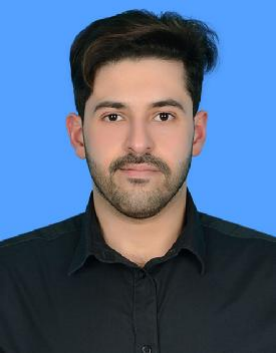


Dr Hasan Majid graduated from the Faculty of Medicine, Kuwait University. He is interested in paediatrics, paediatric emergency medicine, paediatric anaesthesia, and paediatric cardiac anaesthesia. He aspires to join a combined residency training programme in anaesthesiology and paediatrics. Outside of medicine, he enjoys heavy weightlifting and bodybuilding.

**Consent:** Consent was sought from the parents and granted to describe the toddler’s case in a published report.

## Data Availability

The data underlying this article are available in the article and in its online supplementary material.
